# Clinical Significance and Prognosis of Prenatal Diagnosis of Large Umbilical Cord Cysts—A Review Triggered by a Clinical Case

**DOI:** 10.3390/jcm14082564

**Published:** 2025-04-08

**Authors:** Șerban Nastasia, Adina Elena Nenciu, Manuela Cristina Russu, Adrian Valeriu Neacșu, Iuliana Ceaușu, Nicoleta Adelina Achim

**Affiliations:** 1 Department of Obstetrics and Gynecology, Faculty of Medicine, “Carol Davila” University of Medicine and Pharmacy, 020021 Bucharest, Romania; serban_nastasia@yahoo.com (Ș.N.); manuela.russu@umfcd.ro (M.C.R.); adrianvaleriu.neacsu@yahoo.com (A.V.N.); iuliana.ceausu@umfcd.ro (I.C.); nicoleta-adelina.achim@drd.umfcd.ro (N.A.A.); 2 Department of Obstetrics and Gynaecology, “Dr I. Cantacuzno” Hospital, “Carol Davila” University of Medicine and Pharmacy, 020021 Bucharest, Romania

**Keywords:** umbilical cord cyst, first trimester, pregnancy outcomes, true cyst, pseudocyst

## Abstract

**Background/Objectives**: Prevalence of umbilical cord cysts is largely unknown, mainly due to small dimensions and to the fact that only placental and fetal insertion of the umbilical cord are usually assessed. Older studies report a total prevalence of about 3%, regardless of the size. To date, no correlation between the gestational age, the size of the cyst at the moment of diagnosis and pregnancy prognosis can be made. **Methods**: We managed a case of a large umbilical cyst diagnosed in the first trimester. As our experience with this pathology was limited, we performed a systematic review in order to find out the optimal management. **Results**: We report a case of a large umbilical cord cyst that ended in fetal demise at 13 weeks in the absence of any chromosomal and structural anomalies. Our results differ from what was expected from our literature review. Sixteen papers were included in our analysis. According to the selected papers, single cysts are more frequent than multiple cysts (79% single cysts). The mean value of the maximum diameter of the cyst was 32 mm, and there was no difference in number considering the localization of the cyst. Considering the cases in which genetic testing was performed, there were 22.76% modified results. The most frequent genetic disorder was trisomy 18 (53.57% from the modified results). **Conclusions**: Large umbilical cord cysts are correlated with uncertain prognosis. We made the conclusion that large umbilical cord cystic lesions might have an unfavorable prognosis. Although there are case series that have shown an unproblematic evolution of the pregnancy, large umbilical cysts could be associated with increased risk of fetal anomalies and intrauterine fetal death.

## 1. Introduction

Umbilical cord cystic lesions are relatively common ultrasound findings, seen in up to 2.8–3.4% of first-trimester pregnancies [1,2]. When not detected by first-trimester examination (either not performed or not visible), the umbilical cord cysts could be discovered in the second or even third trimester. Advances in ultrasound machines, with Doppler and power Doppler facilities, have increased the rate of awareness and detection of such lesions.

**Figure 1:**
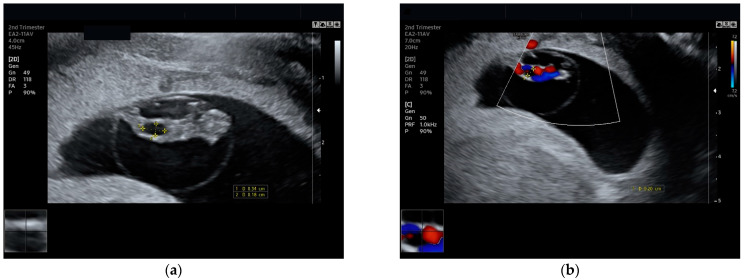
Umbilical cord cyst: (**a**) 0.34/0.18 cm, diagnosed in the first trimester; (**b**) Doppler image showing a small transsonic area, consistent with a small umbilical cyst.

Described as anechoic masses of the umbilical cord [3], these structures are considered a prenatal marker of fetal aneuploidy. With advances in ultrasound machines, the umbilical cord could be visualized once it is well developed; thus, early diagnosis of umbilical cystic lesions is possible starting from 8 to 9 weeks gestational age (Figure 1). This early detection allows for timely counseling and intervention, which can significantly impact prenatal care and the overall health of both mother and baby.

Accordingly, current ISUOG [4] recommendations regarding umbilical cord examination indicate mention of the number of vessels of the cord and the location of the placental cord insertion; however, in particular cases, a further detailed examination of the umbilical cord is warranted. This includes assessing any abnormalities such as cysts, knots or other structural defects that may pose risks during pregnancy or delivery. The objective of this paper is to discuss the characteristics of the umbilical cord cyst in the light of the literature compared to the reported case.

## 2. Materials and Methods

We report on a case of multiple umbilical cord cysts detected in the first trimester of pregnancy in our hospital. The case highlights the importance of thorough ultrasound assessments and the need for individualized management plans, as variations in clinical outcomes can occur.

A systematic review search strategy was applied to PubMed, Web of Science and EMBASE to identify the literature available from January 2010 until September 2024 that reports on fetuses diagnosed with umbilical cord cysts. We used the following keywords for search: ‘umbilical cord cyst’ OR ‘prenatal diagnosis’ OR ‘umbilical anomalies. Inclusion criteria were case reports and case series with the diagnosis of a fetal umbilical cord cyst by ultrasound at any gestational age. Exclusion criteria were the reviews or the diagnosis at birth. No language restrictions were applied. The systematic review was conducted in accordance with the Preferred Reporting Items for Systematic Reviews and Meta-Analyses (PRISMA) [5]. The search yielded a total of 16 studies that met the inclusion criteria, highlighting various clinical outcomes associated with umbilical cord cysts and their implications for prenatal management.

Data collected included gestational age at diagnosis, persistence or resorption of the cyst and associated structural and genetic anomalies. Pregnancy outcomes were also collected.

## 3. Results

### 3.1. Case Report

A 30-year-old G2P0 woman was referred to our clinic at 7 weeks of gestation for pregnancy confirmation. Ultrasound showed a unique embryo, with no other anomalies. No significant medical history could be elicited.

**Figure 2:**
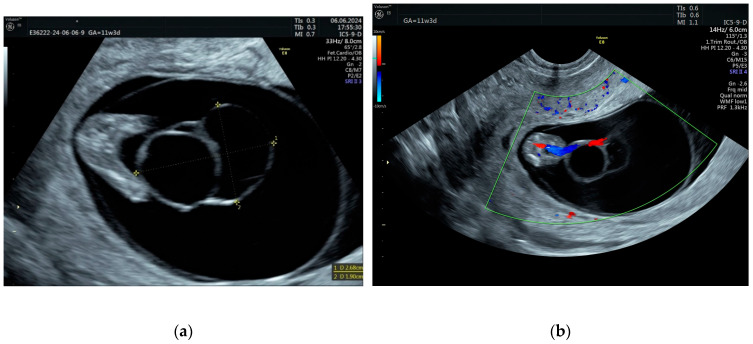
(**a**) Septate cystic lesion, located at the fetal end of the umbilical cord. (**b**) The fetal insertion of the umbilical cord and single umbilical artery are clearly visible.

At 10 weeks of gestation, a septate umbilical cystic lesion was identified, located towards fetal insertion (Figure 2). The dimensions were measured as recommended by Ghezzi [6] from the outer-to-outer border. We considered the lesion to be septate rather than two separate lesions due to the protruding aspect of the left transonic part in the right transonic part of the lesion.

The first-trimester ultrasound scan only identified a soft marker presence (single umbilical artery), with no other fetal anomalies. The first-trimester combined screening test detected a low risk of chromosomal anomalies. As the literature reports the association of two soft markers, umbilical cord cysts and single umbilical artery, to aneuploidy, CVS was proposed to the patient, who denied it. Instead, the patient agreed with NIPT, which showed a very low risk of chromosomal anomalies. From 10 weeks to 13 weeks of gestation, the umbilical cyst lesion had a slowly progressive evolution.

A new ultrasound scan, performed at 13 weeks at the patient’s request, in the absence of symptomatology, showed the death of the fetus. Medical termination of pregnancy was performed. The products of conception were referred to pathology. The genetic studies performed indicate the absence of clinically significant microdeletions or microduplications (arr(X,Y) × 1, (1–22 × 2)).

**Figure 3:**
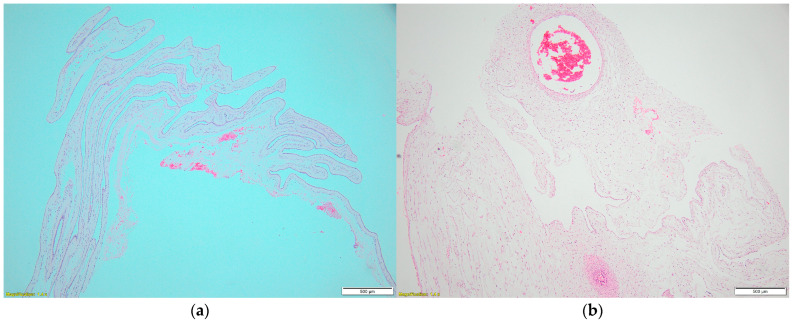
(**a**) Microscopic examination of the placenta confirmed a single umbilical artery. (**b**) The cystic lesion was lined by single-layer cuboid epithelium.

**Figure 4:**
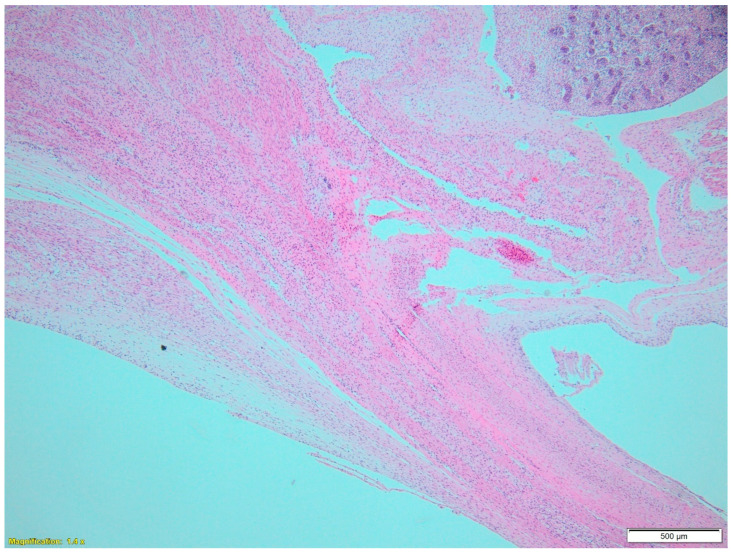
Magnification 1.4. An oblique section at the level of umbilical cord insertion demonstrating normal insertion of the cord on the fetus. The fetal kidney is also visible.

Anatomical assessment feasible at this gestational age on the transverse section of the umbilical cord revealed multiple coils with single-layered epithelial lining (Figure 3a), permitting the diagnosis of a true umbilical cyst. The umbilical artery diameter was markedly reduced, at 57 µm, whereas the umbilical vein diameter was 820 µm, a finding that could explain the fetus’s death (Figure 3b). Pathology revealed normal insertion of the umbilical cord on the fetus (Figure 4).

### 3.2. Narrative Systematic Review

**Figure 5:**
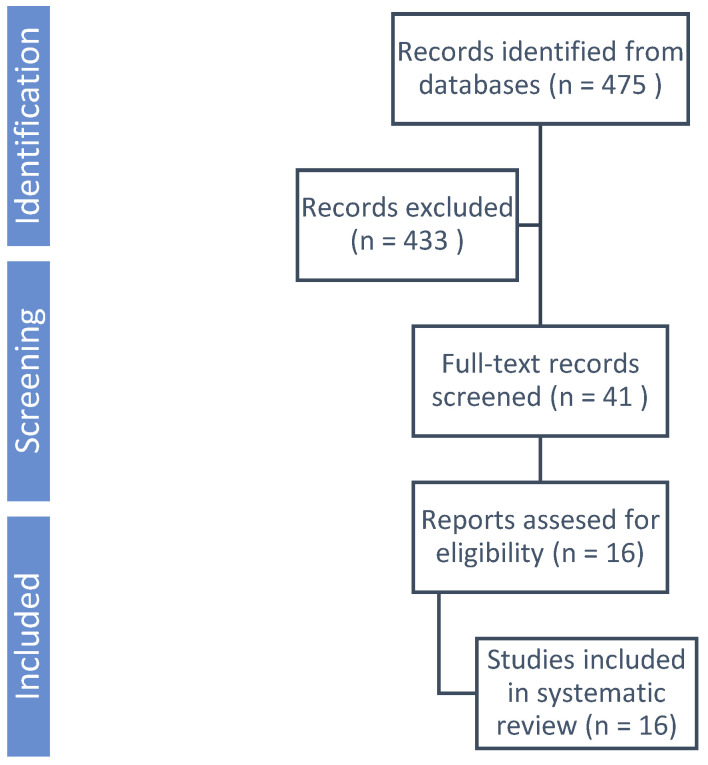
PRISMA flowchart summarizing inclusion of studies in systematic reviews.

The literature search of PubMed, Web of Science and EMBASE databases initially retrieved 475 potentially relevant studies. After evaluation of the title or abstract, 433 were excluded. The remaining 41 studies were reviewed in full text (Figure 5). Overall, 16 studies were included [1,6,7,8,9,10,11,12,13,14,15,16,17,18,19,20]. The findings from these studies provide valuable insights into the prevalence, diagnosis and management of umbilical cord anomalies, emphasizing the importance of early detection and appropriate clinical intervention.

The studies included 250 cases of fetuses diagnosed with umbilical cord cysts during pregnancy. The average maternal age at the time of diagnosis across various studies was reported to be approximately 32.5 years, indicating a trend towards older maternal age in pregnancies with complications such as umbilical cord cysts.

The diagnosis of umbilical cord cyst was made in all trimesters, from 7 weeks to 38 weeks of pregnancy. From the total number of umbilical cysts, 52.4% were diagnosed in the first trimester.

In the 14 studies [1,6,7,8,10,11,12,13,14,15,16,17,18,19] that included this characteristic, 116 cases (79.45%) presented with a single cyst and 30 cases (20.55%) with multiple cysts. In the other two papers [9,20] that included 104 cases, there were no specifications.

Regarding the location of the cyst, three studies did not specify this aspect [1,7,9]. The other studies reported a total number of 53 cases (30.81%) with the cyst localized near the fetal extremity, 56 (32.56%) near the placental extremity and 63 cases (36.63%) with the cyst on a free loop or extended to the center of the umbilical cord.

The largest umbilical cord cyst reported had a diameter of 15 cm [13], followed by 7.5 cm [17], with the smallest cyst detected at 0.9 mm [19]. The overall mean value of the maximum diameter of the cyst was 32 mm, with six studies reporting cysts smaller than 20 mm (mean value of the maximum diameter 11.13 mm) [6,12,14,17,19,20] and the rest of the eight studies including cysts bigger than 20 mm (mean value of the maximum diameter 64.12 mm).

Only two studies [9,20] reported the type of genetic analysis performed (chromosomal microarray analysis and exome sequencing) and applied them to all the cases. In the other studies, the genetic assessment was addressed to the cases in which the cyst was non-isolated. Overall, 123 cases were tested, and 28 (22.76%) had abnormal results. Fifteen cases were diagnosed with trisomy 18 (12.20% from the total tests and 53.57% from the modified results).

Several case reports have documented omphalocele as an isolated finding in fetuses with umbilical cord cysts [15], but in a review of multiple cases, omphalocele was identified in 8 out of 23 reported cases of persistent umbilical cord cysts, highlighting its prevalence in this context [1]. In 49 cases, the patients opted for termination of pregnancy, or the case ended with intrauterine death. There were 201 live births. The data do not indicate a trend towards cesarean delivery in cases with umbilical cord cysts, but exceptions were made when fetal well-being was at risk [11,15]. There were cases where the delivery method was not explicitly stated, but the outcomes suggested that close monitoring often led to surgical births [11]. In 46 cases the cyst resorbed before birth.

This study is limited by the incomplete description of the cysts in previous papers. A comprehensive meta-analysis cannot be performed due to the fact that not all the papers individually present the cases. The absence of a standardized protocol in the evaluation of umbilical cord cysts leads to incomplete information in different papers. As such, a narrative systematic review was performed. The collected data is presented in Appendix A.

## 4. Discussion

Umbilical cystic lesions are common lesions, with an estimated prevalence in the first trimester ranging from 0.4% to 3.4% [2]. Gilboa et al. report a 0.7% prevalence on first-trimester nuchal translucency screening (eight cases in 1080 patients) [17]. The significance of discovering an umbilical cord cystic lesion in the first trimester is still to be established; literature reports vary from no additional risk to the pregnancy to a strong association with chromosomal and structural defects.

The ultrasound examination of umbilical cystic lesions is quite standardized. It should be measured as an outer-to-outer border at the maximum magnification, the yolk sac should be visualized as a separate mass, and Doppler examination should exclude the presence of blood flow at the cystic lesion level.

The fetal risks associated with the presence of umbilical cysts are dependent on many factors, some of them interconnected: number of cysts, association with other fetal anomalies (isolated or not isolated), dimensions of the cysts, progression or regression of the lesion, gestational age at diagnosis and pathological form of the cystic lesion (pseudocyst vs. true cyst).

### 4.1. Number

According to the number, umbilical cord cystic lesions are unique or multiple. Their location varies, from the fetal end of the cord to the free loop and placental insertion location.

Ghezzi’s study, which reported 24 cases, concludes that single and multiple umbilical cord cysts diagnosed in early gestation are two different entities. Although the mean diameter was similar (3.8 (2.1–18) mm vs. 3.05 (2.0–7) mm; *p* = 0.38), they found that multiple cysts were associated with a significantly higher percent of miscarriage (4 out of 6 cases vs. 1 out of 18 cases; relative risk, 7.6; 95% confidence interval, 1.9–30.3) [6]. On the other hand, a single umbilical cord cyst was associated with the delivery of a normal infant without chromosomal abnormalities; in this group, all but one infant were delivered at term.

Therefore, it is considered that single cystic lesions are relatively more common and have the potential to spontaneously resolve, especially when detected early in pregnancy. Multiple cysts, though less frequent, are more often correlated with underlying abnormalities, such as chromosomal anomalies, structural abnormalities and, therefore, adverse pregnancy outcomes [6].

Searching the literature, we were unable to find any reports about septate umbilical cysts and their possible prognostic significance.

### 4.2. Location

The location of the umbilical cysts may play a significant role in determining their clinical outcome. Umbilical cysts could be located in proximity to placental insertion, close to the fetal abdominal insertion or on a free loop. Ross et al. [2] report 29 patients with umbilical cord cysts, out of which fetal abnormalities were found in seven cases (26%).

They concluded that the location of the cysts near the placental or fetal extremity of the cord is more often associated with the presence of fetal anomalies. However, Zangen et al. [15] reports a 10-case series with umbilical cysts detected in the second and third trimesters.

Close placental insertion was detected in eight cases (80%), out of which seven were normal neonates and one neonate had ventricular septal defect and atrial septal defect. There were no cases with umbilical cystic lesions located near fetal extremities.

In the study of Gilboa et al. [17], fetal insertion umbilical cysts were reported in three cases. All three fetuses were abnormal (one case of hypoplastic left heart and one case of trisomy 18, both terminated, and one case of patent urachus, ectopic kidney and umbilical hernia, cured by neonatal surgery).

Taking into account our case, we conclude that fetal insertion of a large umbilical cyst is associated with poor fetal outcome.

### 4.3. Dimension

The relationship between dimension and prognosis is still unknown; most studies concentrate on the association of the presence of cystic lesions and the presence of genetic and structural defects. Some studies report and conclude that only small umbilical cystic lesions, small, isolated cysts, especially in the first trimester, are associated with good prognosis [6].

Hannaford et al. [19] reported on a group of 45 patients with umbilical cysts, diagnosed in the first trimester of pregnancy, with a mean cyst diameter of 3 ± 2.1 mm, compared with 85 matched patients with normal umbilical cords. The association with other abnormal ultrasound findings and fetal death was similar in the two groups. The authors concluded that small umbilical cysts are not associated with poor fetal outcome.

The study of Sepulveda W et al. [3] reports on nine cases of large cystic lesions, more than 2 cm in diameter (eight were single and one was a double cystic lesion), and four cases of small multiple cystic lesions. In the group of the nine large cystic lesions, fetal death occurred in five cases (55%), omphalocele repair was performed in two cases, and a normal neonate was born in two cases (overall, 22% normal cases), suggesting that large cysts might carry an unfavorable prognosis. In their study, median gestational age at diagnosis was 27 weeks (with a range of 15–37), the authors concluding that cystic lesion diagnoses were late findings, cystic masses being detected after 24 weeks of gestation in 8 of the 13 fetuses.

Our current opinion states that large umbilical cysts (larger than 2 cm) might carry an unfavorable fetal prognosis, although there are many small series and case reports on large cystic lesions stating that dimension does not influence the outcome of the pregnancy. Accurate measurement and monitoring of the cyst’s dimensions are essential for assessing potential risks and guiding further diagnostic evaluation [3].

### 4.4. Time of Detection

The moment of detection of the umbilical cord cysts during pregnancy could be correlated with the clinical implications.

#### 4.4.1. Umbilical Cord Cysts Were Detected in the First Trimesters

The diagnosis of the cyst in the first trimester is frequently associated with spontaneous resorption, and it is not usually linked to adverse pregnancy outcomes. Hannaford et al. [19] reported on a group of 45 patients with umbilical cysts, diagnosed in the first trimester of pregnancy, with a mean cyst diameter of 3 ± 2.1 mm, compared with 85 matched patients with normal umbilical cords. The association with other abnormal ultrasound findings and fetal death was similar in the two groups. The authors concluded that umbilical cysts diagnosed in the first trimester of pregnancy are not associated with poor fetal outcome. Gilboa et al. [17] showed that isolated umbilical cysts diagnosed in the first trimester (11 weeks 4 days–12 weeks 2 days) spontaneously disappeared by 23 weeks of gestation, resulting in normal fetuses.

#### 4.4.2. Umbilical Cord Cysts Were Detected in the Second and Third Trimesters

The study of Zangen [15] reported on ten cases of umbilical cysts (out of which nine were detected during the normal first-trimester ultrasound scan) discovered in the second and third trimesters. Out of the nine cases with normal ultrasound in the first trimester, eight normal neonates were delivered, and one neonate had ventricular septal defect and atrial septal defect and was small for gestational age, suggesting the absence of ominous fetal prognosis when cysts are detected in the second trimester. Discovery of an umbilical cystic lesion in the second trimester, along with a normal first-trimester ultrasound scan, probably indicates that second-trimester- and first-trimester-detected lesions are different entities. To note, in this study, no fetal cord insertion umbilical cyst was reported, suggesting that in the second and third trimesters, umbilical cystic lesions occur on a free loop or near placental cord insertion. Pathological examination of the cystic lesions was not reported.

Umbilical cysts persisting into the second or third trimester are more likely to persist until birth. Analyzing 27 cases of cord cysts, Ruiz Campo et al. [1] suggested that persistence of cord cysts is influenced by the time of diagnosis. Out of 11 cases diagnosed in the first trimester, cysts disappeared in 5 cases (45.45%), while cord cysts persisted until birth in 14 out of 16 cases (87.5%) diagnosed in the second and third trimesters. The authors concluded that umbilical cysts diagnosed in the second or third trimesters are more likely to persist until birth.

### 4.5. Pathological Structure

According to pathological structure, umbilical cord cystic lesions are classified as true cysts, lined by epithelium derived from embryonic remnants, and pseudocysts, secondary to edema of Wharton’s jelly, without epithelial lining.

To date, however, ultrasound could not differentiate between true cysts and pseudocysts. To make the issue even more difficult, many cysts and pseudocysts spontaneously resolve during pregnancy, making it impossible to identify the location and to perform pathological examination at delivery [1,17,19].

Differentiation between cysts and pseudocysts is important, as pseudocysts are more frequent and more frequently associated with aneuploidy or congenital defects.

While most studies [1,19] do not report on histology, the study of Sepulveda et al. [3] reported on 13 cases of umbilical cord pseudocysts, of which histological examination was available in 9 cases. The authors propose an ultrasound criterion for differentiating cysts from pseudocysts: at color flow examination, the vessels are widely split apart in true cysts, whereas in pseudocysts the vessels are pushed away together by the mass. In Sepulveda’s study [3], chromosomal anomalies were present in seven cases (7/13, trisomy 18—five cases, trisomy 13—one case, trisomy 21—one case), severe structural defects in two cases (ending with fetal death), omphalocele in two cases (successful surgical repair) and two normal neonates. It is reasonable to conclude that pseudocysts are associated with chromosomal anomalies, whereas a similar correlation could not be proven for true cysts.

### 4.6. Additional Findings

According to the presence of additional ultrasound findings, umbilical cord cystic lesions are isolated or associated with other ultrasound markers (non-isolated), which could be soft markers or frank fetal anomalies. The study of Ghezzi et al. [6] suggests that detection of a single isolated cyst in the first trimester is associated with a favorable pregnancy outcome. In their study, umbilical cysts were isolated in 19 cases (out of 24). In all these 19 cases, the fetuses were structurally normal (18 normal neonates and one PPROM at 22 weeks with delivery of a structurally normal fetus). Association with fetal anomalies occurred in three cases (two cases of trisomy 18, resulting in miscarriage, and one case of obstructive uropathy with TOP). The other two cases of umbilical cord cysts ended in miscarriage at 10 and 11 weeks.

Similar to Ghezzi’s study, Ruiz Campo et al. [1] found a favorable prognosis for isolated cysts, regardless of being unique or multiple.

In their study, Ruiz Campo et al. [1] showed that, when isolated (13 cases out of 27), umbilical cord cysts were associated with good perinatal prognosis in all cases, regardless of the time of diagnosis.

Similarly, Gilboa et al. [17] found that all their five cases of isolated umbilical cord cysts had good neonatal outcomes. The other three cases in their study were non-isolated, being associated with chromosomal anomalies and structural malformations (one case of hypoplastic left heart (TOP—termination of pregnancy), one case of trisomy 18 with cardiac malformation (TOP), and one case of patent urachus and umbilical cord hernia, for which neonatal surgery was performed).

Association between umbilical cord cysts and other soft markers seems not to be correlated with aneuploidy. In a study realized by Liu et al. [9] that included 51 fetuses, 7 presented umbilical cord cysts and soft markers. None of these children had aneuploidy or a pathogenic number of CNVs.

All these studies suggest that umbilical cord cysts do not change the prognosis of the pregnancy by themselves. The presence of umbilical cord cysts could be considered a soft marker, which should trigger the search for associated anomalies. When associated with chromosomal anomalies and gross structural malformations, the prognosis is given by the latter. The most frequent anomalies associated with the presence of umbilical cord cysts are trisomy 18 with associated structural defects, omphalocele, patent urachus or multiple structural defects [10].

### 4.7. Follow-Up

There is no consensus regarding the management of umbilical cord cysts. As a result of the literature review, we proposed the subsequent flowchart for following up on the umbilical cysts.

**Figure 6:**
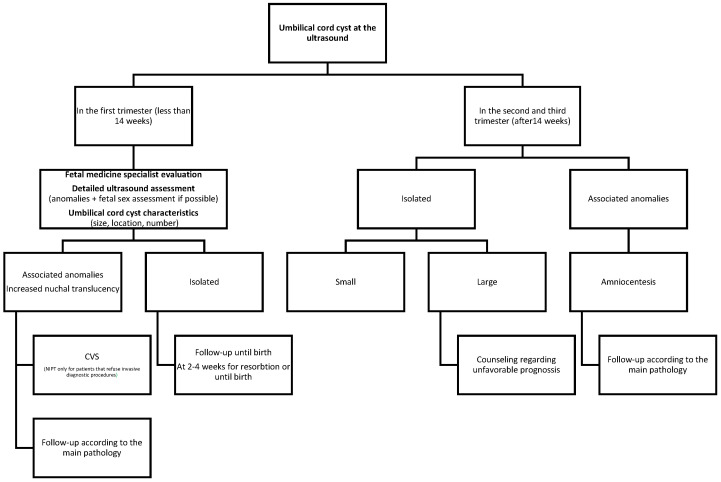
Follow-up of umbilical cyst.

Intrauterine management of umbilical cysts is not reported in the literature, although several mechanisms have been proposed to explain the fetal demise. For early pregnancy, fetal demise could be explained by association with lethal anomalies in non-isolated cases (Figure 6). Mechanical complications associated with the large cysts, like mass-compression effect or umbilical cord hematoma, have been reported by Sepulveda [3]. Reduced diameter of the umbilical artery, especially in cases with a single umbilical artery, could be another mechanism, as suggested by our case.

In term pregnancies, the risk of mechanical complication could not be ignored, so cesarean section (CS) for large umbilical cysts has been proposed [13,21].

Zaigui and Minyue [13] noted that larger or multiple cysts can lead to compression of umbilical vessels. As a consequence, the prognosis is reserved due to the potential umbilical cord rupture and hemorrhage [18]. However, Ruiz Campo [1] reported vaginal delivery in all six cases of persisting isolated cysts in singleton pregnancies.

It is reasonable to presume that there is no indication for cesarean section in structurally normal fetuses diagnosed in early pregnancy with small umbilical cysts, which subsequently disappeared.

## 5. Conclusions

Umbilical cord cysts are correlated with uncertain prognosis. Although there are case series that have shown an unproblematic evolution of the pregnancy, umbilical cysts could be associated with increased risk of fetal anomalies, genetic disorders and intrauterine fetal death.

Large umbilical cysts, isolated or associated with a single umbilical artery, detected in the first trimester, could still carry a high risk of fetal demise. Although as clinicians we would feel the need to perform a shorter-interval follow-up of these patients, the only practical interventions remain the screening for aneuploidies and counselling for possible reserved pregnancy outcomes.

The introduction of the umbilical cord cysts in prenatal assessments could enhance our understanding of their implications, allowing for more informed decision-making and tailored management strategies to support expectant parents through potential challenges.

## Appendix A

**Table A1:** Table including the descriptive data from the 16 studies.

No.	Study	Total Number of Cases Diagnosed During Pregnancy	Number of Cysts	Location	Dimension	Gestational Age at Detection	Other Findings on Ultrasound	Genetic Test	Genetic Test Findings	Follow-Up
**1**	Campo et al., 2017 [1]	27	21 single cysts 6 multiple cysts	Not reported	Not reported	16 weeks (median)	-Omphalocel -ARSA -Pyelectasis bilateral -Megabladder -Multiple structural anomalies: spinal muscular atrophy, arthrogryposis, atrial septal, dysplastic hands, polyhydramnios, bilateral cryptorchiism, brachycephaly -Holoprosencephaly semilobar, HLHS, Omphalocele, single umbilical artery, IUGR severe and early	Fetal karyotyping by invasive technique	-Trisomy 18 in 2 cases -2 cases with partial mole -Edwards syndrome 1 case	15 vaginal deliveries 2 cesarean sections 10 terminations of pregnancy or miscarriages
**2**	Turkmen et al., 2016 [7]	1	Multiple	Not reported	50 × 40 mm	13 weeks	Without other findings	Not specified	Normal results after amniocentesis	Vaginal delivery at 39 weeks
**3**	Hsiung et al., 2024 [8]	1	Single cyst	Fetal pole	80 × 60 mm	36 weeks	-Tetralogy of Fallot (TOF) -Large ventricular septal defect (VSD)	Not reported	Not reported	Vaginal delivery at 36 weeks
**4**	Liu et al., 2022 [9]	49	Not reported	Not reported	Not reported	25.2 weeks (median)	-Hydramnios -Scalp edema -Lateral ventricle broadening -IUGR -Edema of the umbilical cord	G-banding karyotype analysis -Chromosomal microarray analysis -Medical exome sequencing.	-Isolated group: 1 case showed 17p12 microduplication -Non-isolated group: chromosomal abnormalities: 7 cases of trisomy 18, 2 cases of trisomy 13 and 3 microdeletions -Fetus with 15q11.2q13.3 deletion was diagnosed with Prader–Willi syndrome postpartum	Alive births 33 Termiantions 16
**5**	Ghezzi et al., 2003 [6]	24	18 single cysts 6 multiple cysts	Fetal pole 8 Placental pole 6 Central 7 Entire umbilical cord 3	3.8 mm (for single) 3.05 mm (for multiple)	7–14 weeks	Not described	Not specified	-Karyotype in 3 cases -2 cases with trisomy 18	19 live births 5 terminations of pregnancy
**6**	Chien et al., 2021 [10]	1	Single cyst	Fetal pole	30 mm (48 × 45 mm at 22 weeks, disappeared at 28 weeks)	14 weeks	-Patent urachal fistula with continuous urine flow at birth -Bladder herniated out of the fistula	Not reported	Fetal karyotyping was declined	Vaginal delivery at 39 weeks
**7**	Ilhan et al., 2018 [11]	1	Single cyst	Fetal pole	64 × 54 mm	34 weeks	Polyhydramnios	Refused invasive procedures	Postmortem genetic analysis revealed 46, XY karyotype	Cesarean section delivery (fetal distress) Newborn died at 3 days due to perinatal asphyxia
**8**	Svigos et al., 2022 [12]	1	Single cyst	Fetal pole	17 mm × 16 mm × 18 mm (49 × 45 × 47 mm at 35 weeks)	16 weeks	-Homogeneous echogenicity of the cord developed in the third trimester -Umbilical cord edema	Not specified	Normal XX karyotype	Cesarean section delivery Laparoscopic excision of the urachus performed at 11 weeks after delivery
**9**	Zaigui Wu et al., 2020 [13]	23	16 single cysts, 6 multiple Cysts (the rest not described)	Fetal pole 9 Placental pole 9 Central 3 Fetal and central 1	150 mm (1 case) 50 mm (7 cases)	12–38 weeks	-6 cases with multiple malformations -Single umbilical artery -Acromphalus -Kidney absence -Fetal arrhythmia -Fetal cardiac malformation, aortic span -The mitral and tricuspid valves at the same level	Not specified	3 cases with karyotype analysis	6 vaginal deliveries 17 cesarean deliveries
**10**	Bohâlțea et al., 2014 [14]	12	9 single cysts 3 multiple cysts	Fetal pole 2 Placental pole 1 Central 9	3 mm (mean)	11–13 weeks	Absent nasal bone and abnormal ductus venosus	Not specified	1 case of trisomy 18	9 live births with normal fetuses 1 with postnatal surgery
**11**	Zangen et al., 2010 [15]	10	Single cyst	Placental pole 8 Central 2	39 mm (mean)	25 weeks (median)	-Heart malformations and intrauterine growth -Restriction -Polyhydramnios -VSD	Not specified	-4 cases with karyotype -1 case of trisomy 18 -3 normal karyotypes	8 live births 2 terminations of pregnancy
**12**	Aayushi et al., 2018 [16]	1	Single cyst	Placental pole	50 ×50 mm	26 weeks	-Polyhydramnios -No gross congenital malformations	Not reported	The karyotype was not performed due to lack of consent	Cesarean section at 39 weeks
**13**	Gilboa et al., 2011 [17]	8	6 single cysts 2 multiple cysts	Central 4 cases Fetal pole 3 cases Placental pole 1 case	19 mm (between 10 and 38 mm)	11 weeks 6 days	-Hypoplastic left heart -Patent urachus (cystic formation that protruded into the umbilical cord), multiple umbilical cord cysts, crossed ectopic kidney. -Short long bones, atrioventricular canal, echogenic kidneys, clenched hands, single umbilical artery	Not specified	-1 case trisomy 18 -3 cases with normal karyotype; in 4 cases it was not performed	5 healthy neonates 1 surgery for umbilical cord hernia 2 terminations of pregnancy
**14**	Haino et al., 2010 [18]	1	Single cyst	Fetal pole	50 mm (106/47/67 cm at 33 weeks)	21 weeks	-Normal anatomy -Single umbilical artery	Not reported	Parents refused amniocentesis	Cesarean section delivery at 37 weeks
**15**	Hannaford et al., 2013 [19]	45	39 single cysts 6 multiple cysts	Fetal pole 13 Placental pole 8 Central 15 Uncategorized 9	3 ± 2.1 mm (range 0.9 to 5 mm)	8 weeks 3 days	-5 sonographic fetal abnormalities were found in the umbilical cord cyst cohort -1 case local edema over the lower thoracic and lumbar region (resorbed)	Not performed	-On autopsy in the one case with fetal edema—karyotype 46,XY -On the case with fetal demise at 38 weeks, with karyotype 46,XY	45 live births
**16**	Qian et al., 2023 [20]	45	Not specified	Fetal pole 13 Placental pole 22 Central 10	20 mm (mean)	25 weeks 3 days	-Fetal growth restriction -Polyhydramnios/oligohydramnios -Systemic edema and abnormal posture of both lower limbs -Mild left lateral ventriculomegaly and fetal growth restriction	CMA analysis and prenatal whole exome sequencing (WES)	-All patients were tested -5 cases with chromosomal abnormalities -1 case of Trosomy 18 -1 case of 47, XXX -Monosomy of the short arm of chromosome 9 -1 case of pCNV overlapping with 17p12 recurrent region-1 case of X chromosome short arm terminal duplication and chromosome 4 short arm terminal microdeletion (VUS) -3 cases of CNVs of unknown significance	34 live births (4 neonates with postnatal surgery and good prognosis) 11 terminations of pregnancy
